# High-sensitivity troponin in the prognosis of patients hospitalized in intensive care for COVID-19: a Latin American longitudinal cohort study

**DOI:** 10.5935/0103-507X.20220006-en

**Published:** 2022

**Authors:** John Sprockel, Anggie Murcia, Juan Rincon, Katherine Berrio, Marisol Bejarano, Zulima Santofimio, Hellen Cárdenas, Diego Hernández, Jhon Parra

**Affiliations:** 1 Intensive Care Service, Hospital El Tunal, Subred Integrada de Servicios de Salud del Sur - Bogotá, Colombia.; 2 Institute of Research, Fundación Universitaria de Ciencias de la Salud - Bogotá, Colombia.

**Keywords:** COVID-19, Coronavirus infections, Troponin, Reverse transcriptase polymerase chain reaction, Critical care, Mortality

## Abstract

**Objective:**

The current study assessed the prevalence of troponin elevation and its capacity to predict 60day mortality in COVID-19 patients in intensive care.

**Methods:**

A longitudinal prospective single-center study was performed on a cohort of patients in intensive care due to a COVID-19 diagnosis confirmed using real-time test polymerase chain reaction from May to December 2020. A Receiver Operating Characteristic curve was constructed to predict death according to troponin level by calculating the area under the curve and its confidence intervals. A Cox proportional hazards model was generated to report the hazard ratios with confidence intervals of 95% and the p value for its association with 60day mortality.

**Results:**

A total of 296 patients were included with a 51% 60-day mortality rate. Troponin was positive in 39.9% (29.6% *versus* 49.7% in survivors and non-survivors, respectively). An area under the curve of 0.65 was found (95%CI: 0.59 - 0.71) to predict mortality. The Cox univariate model demonstrated a hazard ratio of 1.94 (95%CI: 1.41 - 2.67) and p < 0.001, but this relationship did not remain in the multivariate model, in which the hazard ratio was 1.387 (95%CI: 0.21 - 1.56) and the p value was 0.12.

**Conclusion:**

Troponin elevation is frequently found in patients in intensive care for COVID-19. Although its levels are higher in patients who die, no relationship was found in a multivariate model, which indicates that troponin should not be used as an only prognostic marker for mortality in this population.

## INTRODUCTION

The World Health Organization (WHO) declared coronavirus disease 2019 (COVID-19) a pandemic 1 year ago.^(1)^ COVID-19 is characterized by the presence of outbreaks with a large number of critical cases that overwhelm the health care system.^(2)^ One objective of screening and management is to identify markers to efficiently stratify the individual risk of experiencing adverse results.^(3)^ High-sensitivity troponin was proposed as a prognostic marker for this purpose.^(4)^

Cardiology societies were initially against the customary measurement of troponin due to limited evidence of its utility to make medical decisions and the risk of improper prognostic and therapeutic interventions based on its measurement.^(5,6)^ Intensive care societies have not made any declarations in this respect. A recent meta-analysis showed that remarkable limitations existed in the available evidence, and the justification to measure troponin as a prognostic tool for patients hospitalized for COVID-19 required further research.^(7)^

The present work assessed the prevalence of troponin elevation in patients hospitalized in the intensive care unit (ICU) for COVID-19 to explore its capacity to predict mortality within 60 days.

## METHODS

A single-center longitudinal prospective study of patients who were hospitalized in one of the nine ICUs of the *Hospital el Tunal* in Bogotá, Colombia, for SARSCoV-2/COVID-19 infection confirmed using real-time polymerase chain reaction (RT-PCR) test from nasal swabs between May and December 2020 was performed. The study excluded patients with previous conditions that limited the therapeutic effort (explicitly stated by the patient or family or the presence of comorbidity in an advanced state recorded in the clinical registry), patients who died before 24 hours of hospitalization, patients coming from another ICU where they stayed for more than 72 hours, patients hospitalized for reasons other than COVID-19, and pregnant women.

The *Hospital el Tunal* is a 4th level unit of health services in the Integrated Subnet of Health Services of the South of Bogotá, which has an area of influence of approximately 2 million inhabitants. This unit underwent a restructuring process during the year of the pandemic that included increasing the number of ICU beds from 27 in three units to 103 in nine units. Therefore, this patients in critical condition due to COVID-19 were referred to this center.

The medical records of the hospitalized patients were reviewed to collect clinical data, antecedents, vital signs, and imaging and laboratory results. High-sensitivity troponin I was measured on the Atellica^®^ IM Analyzer (Siemens), and the 99th percentile was set at 0.03ng/mL. Complications (especially cardiovascular complications) and hospital deaths were identified. The vital condition of patients who survived 60 days after hospitalization in the ICU was verified in the national register of deaths system (*Registro Único de Afiliados* - RUAF).

For statistical analyses, qualitative variables are reported as absolute frequencies and percentages, and quantitative variables are summarized as central tendencies and dispersion measurements. For the initial description, a bivariate analysis was performed using Student’s t-test for quantitative variables and the Chi-squared test for qualitative variables. Significant differences occurred at a probability < 0.05. A Receiver Operating Characteristic (ROC) curve was plotted for the prediction of death depending on the different values of high-sensitivity troponin I by calculating the area under the curve (AUC) with its respective confidence interval (CI). Kaplan-Meier survival graphics were plotted to express the probability of death from admission to the ICU through the 60th day between the groups of patients with positive and negative troponin, and comparisons were made using the log-rank test reporting the p value.

The independent hazard factors were identified using a Cox proportional hazards model with a stepwise selection of the variables. A bivariate analysis was performed with time until 60-day death upon admission to the ICU as the dependent variable (outcome), and the variables that did not reach a p value < 0.1 were removed. A multivariate model was generated using the remaining variables, and resampling was performed using ten-fold validation. We report the hazard ratios (HR) with their 95% confidence interval (95%CI) and the p value by applying the Wald test.

The clinical variables assessed as possible prognostic markers upon admission were age, systolic blood pressure, oxygen saturation, hypertension antecedents, diabetes, heart disease, renal disease, smoking, consumption of angiotensin-converting enzyme (ACE) inhibitors or angiotensin receptor blockers (ARBs), number of diseases, leukocytes, neutrophils, lymphocytes, transaminases, total bilirubin, blood urea nitrogen (BUN), creatinine, C-reactive protein, lactate dehydrogenase, high-sensitivity troponin, D dimer, ferritin, and partial pressure of arterial oxygen/fraction of inspired oxygen (PaO_2_/FiO_2_) ratio.

A multivariate logistic regression model was created in which the positivity of high-sensitivity troponin upon admission was taken as the dependent variable. The statistical analyses were performed using R version 4.0.2 and the statistical packages “survival”, “survminer”, “pROC”, “ROCit”, and “Caret”. The committee of ethics and research of the Integrated Subnet of Health Services of the South approved the current study. Informed consent was waived due to the retrospective nature of the study. No funding was used to perform the study.

## RESULTS

**Figure 1:**
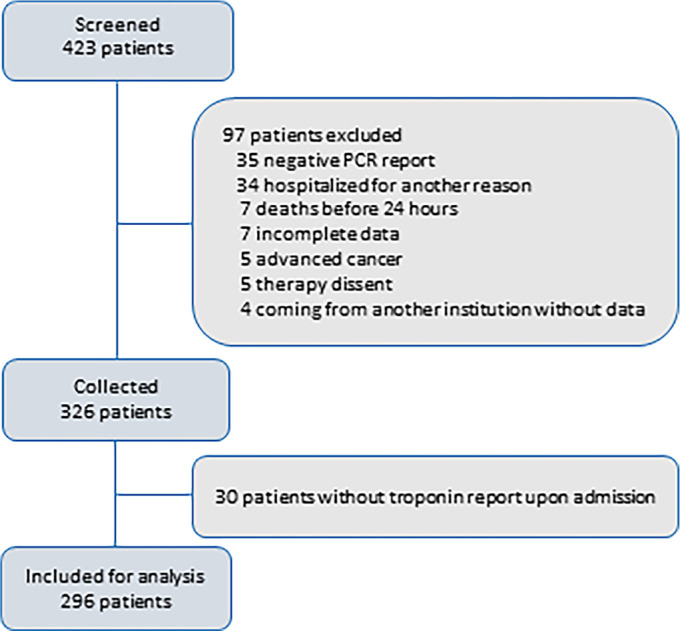
Patients involved in the study. PCR - polymerase chain reaction.

Figure 1 shows the flowchart of the patients from screening to the inclusion of 326 patients in the database.

Troponin levels upon admission to the ICU were available for 296 patients. Table 1 describes the patient characteristics classified according to their vital conditions. A total of 110 (37.2%) patients were female with a median age of 60 years (standard deviation - SD - of 14), and the median duration of symptoms before admission to the ICU was 8.4 (SD of 4.2) days. A total of 81.4% of the patients had at least one comorbidity, with the most frequent being hypertension in 117 (39.5%), diabetes in 65 (21.9%), chronic pulmonary disease in 63 (21.3%), and heart disease in 39 (13.2%). Eighty-four patients were smokers (28.4%), and 124 (46.3%) had obesity. A total of 238 (80.4%) patients had lymphocyte levels < 1,200 cells per µL. The mean value of creatinine was 1.3mg/dL, C-reactive protein 7.8mg/L, ferritin 1,179ng/mL, LDH 1,104 U/L, and D-dimer 4.8µg/mL.

**Table 1:** General characteristics of the population

Characteristics	Population of study (n = 296)		Survivors (n = 145)	Non survivors (n = 151)	p value
Females	110 (37.2)		60 (41.4)	50 (33.1)	0.177
Age (years)	60.0 (14.0)		55.7 (14.5)	64.1 (13.8)	< 0.001
Comorbidities					
Average ± SD	1.8 ± 1.5		1.7 ± 1.4	1.9 ± 1.6	0.256
At least one	241 ± 81.4		118 ± 81.4	123 ± 81.4	1.000
Hypertension	117 ± 39.5		55 ± 37.9	62 ± 41.0	0.666
Diabetes	65 ± 21.9		30 ± 20.7	35 ± 23.2	0.706
Chronic cardiac disease (except hypertension)	39 ± 13.2		13 ± 8.9	26 ± 17.2	0.054
Chronic renal disease	14 ± 4.7		5 ± 3.4	9 ± 5.9	0.457
Smoking	84 ± 28.4		44 ± 30.3	40 ± 26.5	0.825
Chronic lung disease	63 ± 21.3		26 ± 17.9	37 ± 24.5	0.215
Use of ACE inhibitors or ARB	102 ± 34.4		46 ± 31.7	56 ± 37.1	0.182
Obesity*	124 ± 46.3		66 ± 48.9	58 ± 43.6	0.457
Chest pain at presentation	43 ± 14.5		25 ± 17.2	18 ± 11.9	0.257
Duration of the disease before admission to ICU (days)	8.4 ± 4.2		8.5 ± 4.0	8.4 ± 4.4	0.842
Laboratory					
White cell count (×10^3^ cells per µL)	11.9 ± 5.0		11.3 ± 4.4	12.5 ± 5.4	0.040
Lymphocyte count (×10^3^ cells per µL)	0.9 ± 0.7		1.0 ± 0.9	0.8 ± 0.6	0.017
Lymphocytes smaller than 1.2 ×10^3^ cells per µL	238 (80.4)		111 (76.6)	127 (84.1)	0.136
Creatinine (mg/dL)	1.3 ± 1.1		1.1 ± 0.9	1.5 ± 1.3	0.001
High sensitivity C-reactive protein (mg/L)	17.8 ± 15.0		16.2 ± 16.4	19.3 ± 13.4	0.080
Ferritin (ng/mL)	1,179 ± 709		1,061 ± 748	1,304 ± 646	0.005
D-dimer (µg/mL)	4.8 ± 7.0		4.3 ± 7.0	5.3 ± 7.0	0.250
Lactate dehydrogenase (U/L)	1,104 ± 1,174		887 ± 328	1,312 ± 1,586	0.002
High sensitivity troponin I (ng/mL)	0.6 ± 2.1		0.4 ± 2.1	0.7 ± 2.2	< 0.001
Positive high sensitivity cardiac troponin I	118 (39.9)		43 (29.6)	75 (49.7)	0.001

SD - standard deviation; ACE - angiotensin-converting enzyme; ARB - angiotensin receptor blocker; ICU - intensive care unit.

* In 28 patients, there was no information about weight. Results expressed as n (%) or average (standard deviation) or median (standard deviation).

Mortality occurred in 151 cases (51%). Table 2 describes the severity and complications of the patients. Shock was present in 225 (76%) patients, and severe acute respiratory distress syndrome - ARDS - (PaO_2_/FiO_2_ < 100mmHg) was present in 242 (81.8%) patients. A total of 232 (78.4%) patients required invasive respiratory support, and acute renal lesions were present in 153 patients (5.7%). The mean Acute Physiology and Chronic Health Evaluation II (APACHE II) score was 13.1, the Sequential Organ Failure Assessment (SOFA) score was 4.8, and the CURB-65 score was 1.9. There was a trend toward a higher severity score and an increase in complications in patients who died compared to survivors.

**Table 2:** Description of the severity, complications, and possible causes of elevated troponin

Characteristics	Population of study (n = 296)	Survivors (n = 145)	Non survivors (n = 151)	p value
Severity scales				
APACHE II on day 1 of critical illness	13.1 ± 6.6	11.2 ± 5.4	15.0 ± 7.0	< 0.001
SOFA score on day 1 of critical illness	4.8 ± 3.2	4.2 ± 3.0	5.4 ± 3.3	0.001
CURB-65	1.9 ± 1.1	1.5 ± 1.0	2.3 ± 1.1	< 0.001
Organ dysfunction				
Shock	225 (76.0)	92 (63.4)	133 (88.1)	< 0.001
Severe ARDS (PaO2/FiO2: < 100mmHg)	242 (81.8)	108 (74.5)	134 (88.7)	0.002
Received invasive ventilation support	232 (78.4)	89 (61.4)	143 (94.7)	< 0.001
Acute renal lesion	153 (51.7)	45 (31.0)	108 (71.5)	< 0.001
Duration of hospital stay (days)	23.9 ± 16.4	29.2 ± 18.9	18.7 ± 11.5	< 0.001
Cardiovascular complications				
Myocarditis	11 (3.7)	4 (2.8)	7 (4.6)	0.585
Pulmonary embolism	29 (9.8)	11 (7.6)	18 (11.9)	0.290
Acute coronary syndrome	18 (6.1)	6 (4.1)	12 (7.9)	0.260
Acute myocardial injury	116 (39.2)	45 (31.0)	71 (47.0)	0.007

APACHE - Acute Physiology and Chronic Health Evaluation; SOFA - Sequential Organ Failure Assessment; ARDS - acute respiratory distress syndrome; PaO2/FiO2 - pressure of arterial oxygen/fraction of inspired oxygen. Results expressed as average (standard deviation) or n (%).

**Figure 2:**
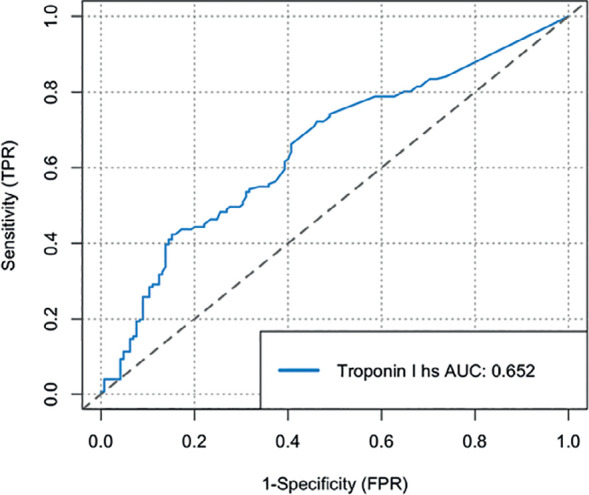
Receiver Operating Characteristic curve of the association between the levels of high-sensitivity troponin I and mortality in COVID-19 patients hospitalized in intensive care. TPR - true positive rate; AUC - area under the curve; FPR - false positive rate.

**Figure 3:**
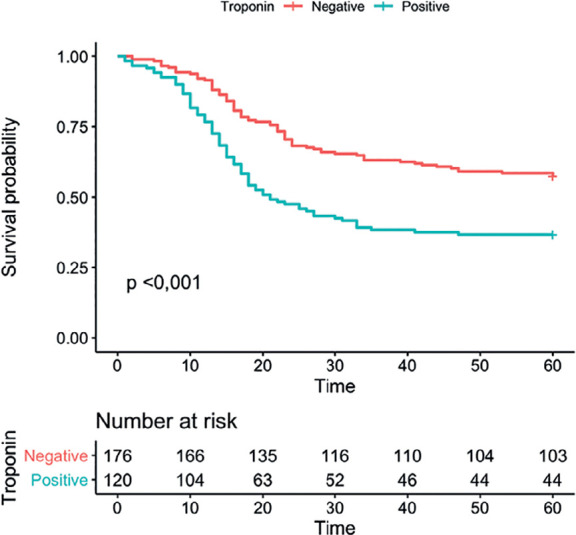
Kaplan-Meier curves of survival analysis for troponin.

The mean level of high-sensitivity troponin I was 0.6 (2.1) ng/ mL, which was positive in 118 patients (39.9%). Troponin was positive in 29.6% of survivors (43/145) and 49.7% of the patients who died (75/151). The possible causes of myocardial elevation based on the clinical history included myocarditis in 11 cases (3.7%), pulmonary embolisms in 29 cases (9.8%), acute coronary syndromes in 18 cases (6.1%) and acute myocardial injury in 116 cases (39.2%). Only this last marker achieved a significant difference between patient who died versus the survivors (Table 2). An area under the ROC curve of 0.65 was found (95%CI 0.59 - 0.71) for the prediction of mortality using troponin (Figure 2). The Kaplan Meier curves are shown in figure 3 and demonstrate higher mortality in patients with positive troponin, with a p value < 0.001 in the log-rank test.

The bivariate analysis showed that troponin had an HR of 1.94 (95%CI 1.41 - 2.67), and p < 0.001. Age, oxygen saturation upon admission, heart disease, leukocyte levels, lymphocytes, LDH, SGPT, SGOT, BUN, creatinine, C-reactive protein, ferritin, and PaO_2_/FiO_2_ showed a relationship with mortality within 60 days in the bivariate analyses, and these factors were included in the multivariate model. Troponin obtained an HR of 1.39 (95%CI 0.21 - 1.56) and a p = 0.12 in the multivariate model. The complete results of the Cox proportional hazards model are shown in table 3). The factors related to the elevation of troponin in the multivariate logistic regression analysis were age (ods ratio - OR - of 1.007; p value = 0.041), the presence of dyspnea (OR of 1.838; p value = 0.046), and oxygen saturation (OR of 0.989; p value = 0.018).

**Table 3:** Complete Cox proportional-hazards model results

Variables	HR	95%CI	p value
High-sensitivity troponin	1.387	0.920 - 2.091	0.118
Oxygen saturation	0.980	0.967 - 0.994	0.006
C-reactive protein	1.011	1.001 - 1.021	0.031
PaO2/FiO2 ratio	0.995	0.991 - 0.999	0.022
Age	1.002	0.987 - 1.017	0.770
Heart disease	1.595	0.884 - 2.877	0.121
Leukocytes	1.000	0.999 - 1.000	0.594
Neutrophils	1.000	0.999 - 1.000	0.457
Lymphocytes	1.000	0.999 - 1.000	0.125
Aspartate aminotransferase	1.000	0.999 - 1.001	0.787
Alanine aminotransferase	1.000	0.999 - 1.001	0.754
Blood urea nitrogen	1.012	0.996 - 1.028	0.133
Creatinine	0.876	0.674 - 1.138	0.322
Lactate dehydrogenase	1.000	0.999 - 1.000	0.876
Ferritin	1.000	0.999 - 1.000	0.129

HR - hazard ratio; 95%CI - 95% confidence interval; PaO_2_/FiO_2_ - pressure of arterial oxygen/fraction of inspired oxygen.

## DISCUSSION

The damage caused by COVID-19 primarily occurs in the lung tissue but may involve other tissues directly or indirectly, particularly in more critical patients.^(8)^ Myocardial involvement is identified by the presence of high levels of troponin, which has been related to the evolution toward more severe presentations of the disease and death.^(9)^ The proposed mechanism of COVID-19 damage is the excision of the viral S protein by a serine protease, which allows attachment to the angiotensinconverting enzyme 2 (ACE2) and entry into macrophages, perivascular pericytes and cardiomyocytes. Entry of the virus induces myocardial dysfunction and damage, endothelial dysfunction, microvasculature, plaque instability, and myocardial infarction.^(10)^

Myocardial lesions are frequently identified in patients hospitalized for COVID-19^(11)^ and are reported in 7% to 20% of this population.^(12)^ A recent systematic review found a combined prevalence of positive troponin of 38% (95%CI 28.2 - 48.3%) in seven studies that included 814 patients hospitalized in intensive care,^(7)^ which is very similar to the present study (39.9%). Individual studies show variability that ranges from 27%^(8)^ to 51%.^(13)^ Notably, the prevalence in our study is lower than previous reports in patients with ARDS, in which troponin positivity was documented in 56% of the cases,^(14)^ and the variation in troponin levels over time was associated with mortality in this population.^(15)^

A multivariate logistic regression analysis that included 670 hospitalized patients identified the comorbidities of an elderly age (e.g., hypertension, coronary disease, chronic renal failure, and chronic obstructive lung disease) and the reactive elevated C protein as myocardial lesion predictors.^(16)^ Except for age, the remaining factors differed from the factors obtained for the target population in the present study, which focused on critical patients. The area under the ROC curve for initial cardiac troponin I to predict in-hospital mortality was 0.92 (95%CI 0.87 - 0.96), which differs widely in its capacity compared to the results obtained in the present work (AUC = 0.65).

Several studies investigated the relationship between troponin and mortality in patients hospitalized in general hospital wards. A preliminary study from Zou et al. found a strong association with mortality in a univariate analysis, which did not remain in the multivariate analysis.^(17)^ Shi et al. found a positive association with mortality, documenting an HR of 4.3 (95%CI 1.9 - 9.5) using a Cox regression model.^(9)^ A meta-analysis of 11 studies (13,889 patients) that performed a multivariate analysis of the association of troponin upon admission with mortality found a relative HR of 2.7 (95%CI 2.1 - 3.5) with substantial heterogeneity and a possible publication bias.^(7)^

Studies that included intensive care patients and the use of troponin as a prognostic marker of mortality via the adjustment of covariates showed contradictory results. Azoulay et al. included 370 patients in whom troponin was negative in 68% of the survivors compared to 41% for the patients who died and obtained an HR of 0.48 (95%CI 0.31 - 0.75) for its association with mortality within 28 days in the multivariate model.^(18)^ Xu et al. examined the first 239 patients hospitalized in the ICU in Wuham (China) and identified that myocardial lesions occurred more frequently in patients who died (55.1% versus 23.9%; p < 0.001), but the multivariate model did not confirm this association (HR 0.88; 95%CI 0.57 - 1.34; p value = 0.542).^(19)^ The first report by the networked open European registry RISC-19-ICU, which included 639 critical patients, revealed that troponin was higher in patients who died (HR of 2.09; 95%CI 1.50 - 2.91), but it was not significant in the Cox multivariate model.^(20)^ Xie et al. performed another multicenter study in China that included 733 patients and found that troponin I was more than ten times the 99th percentile in the multivariate model and had an HR of 1.45 (95%CI 1.05 - 2.01; p-value = 0.025) for the prediction of mortality within 28 days.^(21)^

This has not been the only negative report for the association of troponin with adverse outcomes in COVID-19, as documented in the RISC-19-ICU study already described;^(20)^ this fact could be explained by the different techniques applied and the variables included in the model (e.g., their quantity, quality or coding). The strategies for the identification of prognostic markers have significant limitations, as stated in Pepe et al.^(22)^

The present study had several limitations. The data were obtained from a single-center, and the results may not be suitable for extrapolation to other populations. The retrospective nature of the study limits interpretation of the findings. Another aspect that limits the analysis is that a systematic evaluation of the cause of the elevation of troponin, such as electrocardiograms, echocardiograms or pulmonary angiographic tomography, were not available to determine the cause due to the retrospective data collection from the clinical record.

The strengths of the study include the acceptable number of outcomes in this cohort to perform the association analysis, with a large intensive care cohort of Latin American patients who also had a follow-up through the 60th day after admission to the ICU. The results of the present work contribute to the evidence of the role of troponin as a prognostic marker in this population.

## CONCLUSION

There was a high prevalence of elevated high-sensitivity troponin I in patients hospitalized in intensive care, and troponin levels were higher in patients who died. The area under the ROC curve showed a moderate power of prediction of 60-day mortality. The difference observed was significant in the univariate analysis, but this association did not remain in the multivariate model. Therefore, troponin should not be used as the only prognostic marker for mortality in this population.
